# Bioprocessing of Human Mesenchymal Stem Cells: From Planar Culture to Microcarrier-Based Bioreactors

**DOI:** 10.3390/bioengineering8070096

**Published:** 2021-07-07

**Authors:** Ang-Chen Tsai, Christina A. Pacak

**Affiliations:** 1 Department of Pediatrics, University of Florida, Gainesville, FL 32603, USA; 2 Department of Neurology, University of Minnesota, Minneapolis, MN 55455, USA

**Keywords:** human mesenchymal stem cell, hMSC, bioprocess, bioreactor, microcarrier, cell expansion, cell counting

## Abstract

Human mesenchymal stem cells (hMSCs) have demonstrated great potential to be used as therapies for many types of diseases. Due to their immunoprivileged status, allogeneic hMSCs therapies are particularly attractive options and methodologies to improve their scaling and manufacturing are needed. Microcarrier-based bioreactor systems provide higher volumetric hMSC production in automated closed systems than conventional planar cultures. However, more sophisticated bioprocesses are necessary to successfully convert from planar culture to microcarriers. This article summarizes key steps involved in the planar culture to microcarrier hMSC manufacturing scheme, from seed train, inoculation, expansion and harvest. Important bioreactor parameters, such as temperature, pH, dissolved oxygen (DO), mixing, feeding strategies and cell counting techniques, are also discussed.

## 1. Introduction

Human mesenchymal stem cells (hMSCs) have been considered promising for therapeutic development to treat many types of diseases. Most of these disorders (including neurological, bone and joint and cardiovascular diseases) lack any disease altering treatment options [1]. In addition to classic studies that demonstrated hMSCs multilineage differentiation capabilities and their ability to home or migrate to injury sites, more recent studies have shown their ability to perform paracrine secretion and immunomodulatory effects [2,3]. Since the first case was reported in 1995, hMSCs have been increasingly tested in the clinic with more than 1100 clinical trials registered at clinicaltrials.gov to date [4]. Among these, the numbers of trials using autologous and allogeneic hMSC were comparable until 2015, after which the number of allogeneic hMSC trials surpassed autologous hMSC trials [5]. This trend likely results from the benefits of immunomodulation which allows hMSCs to be immunoprivileged and thus less able to trigger the transplantation-induced immune rejection that is always a key concern in cell-based therapies [6,7,8]. An additional benefit is the reduced cost of manufacturing associated with allogenic cell products: ($1490–$1830/dose) compared to autologous ones ($3630–$4890/dose) based on some assumptions [7]. Thus far, 9 out of 13 approved hMSC-based products use allogeneic cells [9]. Based upon clinical trial data, transplantation efficacy relies on a specific range quantity of qualified cells (a typical dose ranges from 70 to 190 million cells with a median of 100 million cells) successfully administered to the recipients. Achieving sufficient cell numbers from originating cells requires large in vitro expansion schemes [5]. The need for efficient systems to consistently amplify large quantities of high quality hMSCs for upcoming late-phase clinical trials and future commercialization continues to grow [10].

For clear economic reasons, efficient scaling-up of manufacturing is a desirable endeavor to increase feasibility of allogeneic hMSC therapies [11,12]. To expand anchorage-dependent cells such as hMSCs in scaled-up manufacturing, efficient exploitation of the accessible surface determines the maximum volumetric productivity and the manufacturing lot size. The most common contemporary approach to manufacture hMSCs relies upon multi-layer vessels that are designed as stacked layers within one chamber to enable handling of all layers at the same time to provide higher space efficiency and a more economic bioprocess than regular T-flasks. Despite their improvements over traditional cultureware, hMSC expansion in these multi-layer vessels just barely hits the threshold cell numbers needed for clinical relevance [13]. To further scale up the process, microcarrier systems offer a much higher surface-to-volume ratio that maximizes space efficiency. Microcarriers have been used in various iterations for human cell culture since 1967 [14]. Pioneer studies demonstrated hMSC expansion on microcarriers in fully controllable liter-scale bioreactors [15,16,17,18]. Thanks to recent increased interest in manufacturing economics to expand the use of hMSCs from research and development stages to commercial manufacturing for standard therapies [19], industrial empirical studies have reported that microcarrier bioreactor cultures can reach nearly 80-fold volumetric cell production while only occupying 10% of current Good Manufacturing Practice (cGMP) manufacturing space with a reduced manufacturing cost ($0.044/cm^2^) as compared to multi-layer vessel cultures ($0.061/cm^2^) [20,21]. For these reasons, the development of hMSC production in microcarrier bioreactor systems represents a valuable advancement for the field [13,22,23]. Taken together, microcarrier-based bioreactor systems are an up-and-coming approach to profitably manufacture hMSC-based products for commercialization.

Even though microcarrier bioreactor cultures are theoretically simply an amended approach to generate sufficient cells for transplantation, the innate manufacturing processes are significantly different from those of standard, well established planar cultures. For example, microcarrier culture requires dynamic flow to suspend microcarriers and homogenize bulk medium. This enables mass transfer of oxygen, nutrients and cell metabolites predominantly through convection which is much more efficient than the diffusion that is required in planar culture. Concurrently, the dynamic flow alone induces shear stress on cells and the frequency of cell-bead and cell-cell collisions combined with the sensitivity of hMSCs to mechanical and physical forces, are all thought to influence cell growth and quality [24,25,26]. Another important difference is that the cell growth surfaces in microcarrier cultures are quite different from the flat surfaces that T-flasks or petri dishes provide. In planar culture, cells instinctively adhere the flat surface due to the gravity within a couple of hours after inoculation; contrarily, sufficient cell attachment on microcarriers is determined by cell-bead collisions in addition to adhesion and may take more than one day. Furthermore, in contrast with the integral surface in planar cultures, the surface that microcarriers provide is isolated, which prevents bead to bead cell migration. In other words, the observation that cells primarily propagate and proliferate within an individual microcarrier underscores the importance of the initial colonization efficiency that is not a crucial factor in 2D planar culture. Incomplete liquid removal is another hurdle for microcarrier-based bioprocessing. For instance, a complete medium change is a simple routine for T-flasks or multi-layer vessels. In contrast, a typical medium change volume for microcarrier-based cultures is approximately 25% to 50% (Table 1), depending on the microcarrier bed depth once all microcarriers have settled to the bottom of the bioreactor. A related hurdle that complicates multiple steps during the cell harvest processes is incomplete liquid removal. Consequently, each step of the microcarrier bioreactor cell harvesting process including wash, cell detachment, microcarrier filtration and cell suspension concentration involves special considerations and corresponding strategies to maximize liquid removal without negatively impacting cell quality or yields. Moreover, scaling-up of microcarrier bioreactor processes requires specialized equipment at very large scales. As typical cell strainers can only pass tens of milliliters of cell suspension and laboratory-scale centrifuges are limited to liter volumes, neither are feasible options for efficient harvesting of bioreactor volumes, which can be scaled up to tens or even hundreds of liters of cell suspension. Thus, although these newly evolved bioreactor process parameters improve the scaling-up process at the cell growth stage, they create additional downstream volume-related challenges for microcarrier-based hMSC production. Further improvements in downstream equipment design are needed to fully enable successful process development.

The advantages of microcarrier suspension in bioreactors, such as the easily scalable vessel design, homogeneous nutrition and oxygen access, the real-time on-line/off-line monitoring of cells and medium, versatile operations for feeding strategies (i.e., batch, fed-batch and perfusion) etc., are evident, processes (dynamic cell attachment, distinctive expansion and in-situ harvest) must be adapted from the original methodologies of planar cultures to the needs of microcarrier suspension cultures. The implementation of process analytical technology (PAT) is recommended by the United States Food and Drug Administration (FDA) to clarify processes that aim to facilitate innovation and risk-based regulatory decisions in development, manufacturing and quality assurance [51]. Screening critical process parameters and understanding the influence of the deviated processes on the final product quality are crucial. Our previous studies have delineated the alteration of hMSC properties and therapeutic potencies stemming from micro-environmental differences between planar culture to microcarriers, including the discontinuous surface, convex curvature, microcarrier rigidity, shear stress, collision and aggregation [52,53]. Here, we provide a comprehensive review of bioprocessing changes needed when manufacturing hMSCs in microcarrier-based bioreactors. The process for large scale preparations is also discussed.

## 2. Bioprocessing for Microcarrier-Based hMSC Manufacturing

Scalability is the greatest advantage of microcarrier-based suspension culture over conventional planar culture. The first challenge in developing microcarrier-based hMSC manufacturing is microcarrier selection. Many types of microcarriers have been used in hMSC production in bioreactor systems, including polystyrene, gelatin, dextran and others [23,54]. Evaluations of microcarrier screening for hMSC production have been reported in several studies, primarily regarding three key culture steps: (1) adhesion efficiency, (2) growth rate and cell expansion and (3) ease of detachment [55,56,57]. Rafiq et al. systematically evaluated 13 commercial microcarriers for bone marrow-derived hMSC production in bioreactors and determined that Plastic microcarriers were the most optimal [56]. Loubière et al. compared 5 commercial microcarriers for umbilical cord-derived hMSC expansion and demonstrated that although Cytodex-1 demonstrated higher cell attachment efficiency and better cell expansion performance, Star-Plus and Plastic-Plus were found to be better compromises, for the orbital and the mechanical agitation modes, respectively [55]. Leber et al. screened 6 commercial microcarriers for propagation of bone marrow-derived hMSCs and the immortalized cell line hMSC-TERT in serum containing or chemical defined medium (Stem Cell 1). Their results suggested that Glass-Coated microcarriers supported hMSC-TERT growth and bead-to-bead transfer in serum containing medium whereas Enhanced Attachment microcarriers enhanced cell growth in chemical defined medium [57]. Taken together, microcarrier selection may vary with different systems and specific cell types.

Even though there is a diverse array of microcarrier options, they share a similar geometric round shape with a range of 100–300 µm in diameter. Within this range, microcarriers contribute sufficient volumetric surface without sacrificing cell attachment and proliferation support. Microcarriers designed to maximize space coupled with bioreactor systems that precisely control the culture environment are particularly promising. For example, one liter culture of Synthemax II microcarriers with a loading density of 16 g/L provides 5760 cm^2^ which approaches the 6360 cm^2^ that a CellSTACK^®^-10 provides [33,34]. Further improvements in bioprocesses related to seeding, feeding and harvesting cultures will be discussed to overcome the challenges associated with the use of microcarrier-based bioreactor systems.

### 2.1. Seed Train

In large-scale hMSC manufacturing (>50 L), direct inoculation of cells from cell bank vials into the production-scale bioreactors is unrealistic and uneconomical. Thus, thawing cells from frozen vials, serial passaging through seed trains to sequentially larger and higher-volume vessels is a necessary stage of the manufacturing process [9]. In order to obtain adequate cells for bioreactor inoculum, seed trains processes have been developed for the efficient generation of viable cells from one scale to a subsequently larger one to ultimately inoculate a production-scale bioreactor [58]. Each seed train must reliably generate a predetermined cell number required for the inoculation while consistently maintaining cell quality. Accordingly, beyond assurance of the fold expansion, the quality of seed trains ultimately determines the downstream success of hMSC production. The most important factor that impacts seed-train quality optimization is the time frame as hMSC overgrowth can result in cell-cell contact inhibition and extended passaging processes impose undue cell stress that negatively impacts downstream stages of the pipeline.

Most previous studies used planar culture for hMSC manufacturing due to its relative simplicity with shorter processing times, less variation in cell expansion and consistent cell number yields. However, if the targeting inoculum is above the scale that planar cultures can achieve, microcarrier cultures can also be incorporated into seed trains [32,59]. An intriguing alternative that simplifies the process, is the direct transfer of seeded microcarriers from the seed train stage into the scaled-up bioreactor with fresh medium and empty microcarriers [37,46]. This approach requires satisfactory cell migration from seeded beads to unseeded beads but skips the passaging processes, such as enzymatic treatment, mechanical force detachment, centrifugation and resuspension of cell pellets. Several studies also demonstrated the practicality of inoculum preparation with freshly thawed cells [26,28,30], thus demonstrating that complete replacement of planar cultures with microcarriers is feasible. Due to the increased complexity of the passaging processes for microcarrier cultures, planar culture is preferred for seed trains.

### 2.2. Inoculation

For bulk systems, more effort is required to establish culture conditions at the setpoint. Prior to cell inoculation, microcarriers should be thoroughly hydrated and balanced with the medium, and the entire culture environment should be stabilized at an optimized temperature, pH and DO, to enable immediate resumption of culture conditions following the necessary disruptions caused by inoculation. Upon cell inoculation, hMSCs tend to adhere to a surface as they do in planar culture. However, cell attachment on microcarriers is more difficult than the adhesion in planar culture, not only due to the dynamic environment but also because of the curved microcarrier surfaces. Therefore, most process development studies initially focus on achieving high cell attachment efficiencies [56,60]. Attachment efficiency can be determined by the ratio of adherent cells on microcarriers and/or those cells that remain suspended in the supernatant as compared to the initial inoculation number, assuming no cell growth or death during the analysis timeframe [27,36]. 18 to 24 h are the accepted timeframe within which cell attachment is easily measurable and cell proliferation can be ignored.

Beyond cell attachment, colonization efficiency is also important. Due to the discontinuity of the accessible expansion surface provided by individual microcarriers’ surfaces, cell propagation is restricted within the colonized microcarrier, regardless of bead-to-bead transfer. Colonization efficiency determines the microcarrier surface utilization rate which may affect the final cell yield. Generally speaking, cell colonization efficiency of microcarriers is estimated by Poisson Distribution based upon cell-to-bead ratios and assumes that events of cell-microcarrier collision and successful attachment are random, independent and constant [61,62]. For example, the theoretical prediction of occupied microcarriers at a cell-to-bead ratio of 3 is 95% while a cell-to-bead ratio of 6 is 99.8%. Therefore, cell-to-bead ratios from 3 to 6 are commonly used (Table 1). Practically, the microcarrier amount is chosen by the required surface area necessary to reach the target cell number and the cell seeding density is calculated according to the previous planar culture experience and normally is within 3000 to 5000 cells/cm^2^ (Table 1). To increase the cell-to-bead ratio for higher colonization efficiency, increasing the inoculation cell number is usually more advantageous than the reduction of loaded microcarriers. Even though colonization efficiency and cell attachment are equally important, few studies have fully evaluated the impacts of both [33,38].

Intermittent agitation has been implemented to enhance cell attachment in microcarrier suspension cultures. Yuan et al.’s experimental results in spinner flasks showed that 24 h after cell inoculation the use of intermittent agitation (3 min agitation at 60 rpm followed by no agitation for 27 min) enhanced cell attachment efficiency 1.5 to 2-fold more than continuous agitation at 60 rpm [59]. For intermittent agitation, the static period enhances cell attachment while the agitation period increases cell colonization. If the static period is too long, increased cell attachment leads to higher growth in the beginning but likely not the highest final cell yield due to the limited utilization of microcarriers. Furthermore, because the viability of anchorage-dependent hMSCs decreases the longer they remain unattached to a surface the balance of the intermittent agitation mode must be carefully optimized to be scale-appropriate.

Several methods have been developed to boost cell attachment without compromising colonization efficiency. Successful hMSC adhesion requires both cell-microcarrier collision and sufficient binding of the cell to the microcarrier during the collision. One approach targeting this is to increase the probability of collision. A simple way to increase the collision probability is to reduce the working volume during the cell attachment phase [27,38]. Increased agitation speed can also improve collision frequency; however, reduced contact time during the collision and intensified fluid shear stress due to the higher kinetic energy may ultimately reduce the successful adhesion [63,64]. Advances in biomaterial biotechnology also improved binding, such as microcarriers coated by proteins or positive charge enhancing cell attachment [35,42,65]. For example, Shekaran et al. examined the impact of fibronectin and/or poly-l-lysine-coated polycaprolactone microcarriers on hMSC attachment efficiency and found that sequential coatings of fibronectin and poly-l-lysine improved total cell attachment around 69.4% to 78.3% [65]. In addition, the influence of media has also been studied. Reduced cell attachment and increased lag phases have been observed in serum- and xeno-free medium as compared to serum-supplemented medium, demonstrating the essential role of serum in cell attachment [66]. In another study, Gadelorge et al. successfully used 0.5% platelet lysate (PL) to enhance cell attachment for the first 24 h instead of 5% PL which was used in expansion phase [41]. Despite some advances, strategies to increase the efficiency of cell attachment in microcarrier suspension cultures remains a challenge.

### 2.3. Cell Expansion

After hMSCs securely adhere to microcarriers, cells begin to expand. To maximize cell yields, an optimized environment must be maintained. In planar culture, temperature is controlled by the incubator, pH remains balanced by the CO2 concentration with the sodium bicarbonate in the medium, oxygen is supplied by ambient air and nearly complete medium changes provide nutrient replacement and waste removal. In microcarrier culture, these parameters are all controlled by bioreactor systems that maintain the optimized temperature, pH, dissolved oxygen (DO), perform controlled agitation, nutrient feeding (e.g., glucose and glutamine) and waste removal (e.g., lactate and ammonia) [67]. In contrast to planar cultures that require feeding only once or twice per batch, microcarrier-based bioreactor cultures need more sophisticated bioprocesses to enable real-time monitoring and control with the appropriate feeding strategies to dynamically maintain the optimal cell growth. To increase the ultimate yield, cell culture systems aim to expand working volumes and cell concentrations and therefore require the development of feeding regimes that support a maximum fold increase in cell number.

#### 2.3.1. Mixing (Agitation and Rocking)

Culturing cells on microcarriers in suspension cultures requires some form of mixing. A previous report summarized the influences of mixing-induced fluid shear stress and dynamic environmentally-caused collisions and consequent aggregation on hMSCs therapeutic potencies. The study’s conclusion was to retain the mixing as low as required speeds that merely satisfy the mass transfer and avoid microcarrier accumulation in the dead zone [53]. Stirred-tank bioreactors have been widely used in microcarrier suspension culture. The minimum required agitation speed for maintaining microcarriers in suspension can be easily estimated by visualization [17,42] or measured by particle image velocimetry [28,68]. N_S1_ and N_S1u_ are commonly used criterion that represent the impeller speed [28]. N_S1_ is defined as the threshold agitation to fully suspend all microcarriers but does not ensure a homogenous microcarrier dispersion [69]. N_S1u_ is defined as the minimal agitation necessary to ensure there are no microcarriers at rest even they are in contact with the bottom of the vessel [70]. When considering scale-up manufacturing of hMSCs using microcarrier-based bioreactor systems, an important mixing factor is volumetric energy dissipation. This indicates the power input per unit volume needed to maintain microcarriers in suspension and is proportional to agitation speed [28,68]. Therefore, the agitation speed typically increases along with the culture time to offset the heavier microcarriers loaded with propagating cells, larger working volume or multi-microcarrier aggregation [28,31,36,45,48]. Another factor related to mixing is the impeller tip speed, at the point of which is the highest shear stress in the entire bioreactor, and thus the most severe potential damage to cells. Thus, the dimension and geometry of the impeller are likely critical for viability [28]. It may be the case that a small percentage of cells become exposed to the localized high shear stress but the average shear stress exposure remains low [26].

To bypass the potential for shear stress, other bioreactor types, including fixed-bed bioreactors, hollow fiber bioreactors and wave motion bioreactors, have also been used to expand hMSCs [26,44,71,72]. Microcarrier suspensions in wave bioreactors have demonstrated significantly lower shear stress [64,73]. However, challenges may occur during in-situ cell harvest from these systems due to the required higher shear stress to detach cells from microcarriers. Specific types of microcarriers, such as dissolvable microcarriers, represent an option to overcome this issue.

#### 2.3.2. Control System (Temperature and pH and DO)

The optimal temperature set-point is 37 °C for hMSC culture regardless of the type of culture. Bioreactors are typically equipped with a temperature control unit which receives the signals from the temperature sensors and controls the ability to heat or cool the system as needed. There are two ways to regulate the temperature in bioreactor culture. One is to maintain the temperature of the water jacket of the bioreactor [40,71] or the metal plate attached to the bioreactor bag [44,74] while the other is directly to control the temperature of the bulk culture by measuring the temperature with a thermocouple or a temperature sensor and then to warm the bioreactor with a heating blanket or pad [36,75]. The former may have some deviations due to the indirect measurement while the latter requires more attention to understand the relative locations of the temperature sensor and the heating source. While heat transfer and the temperature distribution should be homogeneous throughout a culture in agitation mode, this may not be the case when the culture is not under agitation (e.g., when settling microcarriers down for a medium change). Any temperature gradient caused by inefficient heat transfer throughout a culture could result in overheating a particular area. Therefore, when the agitation is off, the heating function should be off as well.

The pH value in planar culture is balanced by the 5% CO_2_ concentration in the incubator and the NaHCO₃ in the medium. Even though there is no direct control, the high surface-to-volume ratio for efficient gas mass transfer easily maintains pH values within a certain range in planar culture. On the contrary, dynamic microcarrier-based cultures containing high cell densities at harvest require more specific controls on pH values. As shown in Table 1, the pH setpoint falls between 7 to 7.5 and the pH can be controlled by diluted base (e.g., NaOH, NaHCO_3_) and diluted acid (e.g., H_2_SO_4_) or CO_2_ gas [27,35,36,40,43]. Two studies have successfully maintained pH merely through the use of 5% CO_2_ gas [15,46]. Even though in-line pH probes must be calibrated before each use, off-line calibration (i.e., samples from culture immediately measured by off-line pH meters) enhances the measurement accuracy during cell expansion, especially for long-term cultures.

Dissolved oxygen (DO) is an essential factor for maintaining metabolic activities and cell growth. There are two ways to supply oxygen to bioreactor culture, overlay and sparging. For overlay aeration, gas is continuously pumped into the headspace to adjust the partial pressure that affects dissolved gas in the medium. The concept is that pumping pure oxygen into the overlay can expedite oxygen dissolvability. Sparging supplies oxygen into the medium from the bottom through generating small bubbles that increase the gas-liquid (oxygen-medium) interfacial surface and gas residency time [76]. Due to the low oxygen demands of hMSCs (1.2–3.8 × 10^−17^ mol oxygen s^−1^ cell^−1^) compared to bacteria and yeasts, the DO can often be maintained by overlay aeration [77,78]. Thus far, few studies have implemented sparging in microcarrier-based hMSC production [29,31]. However, if the cell density reaches a certain point and the gas-liquid surface-to-volume ratio is too low, sparging can be applied. One consideration is that because sparging has the potential to increase the shear on cells and may cause foaming at the gas-liquid interface, addition of an anti-foaming agent may be required.

Oxygen transfer rate, to indicate the proficiency of the oxygen mass transfer in a bioreactor, is determined by the interfacial area, mass transfer coefficient and oxygen concentration gradient [79]. The interfacial area is dictated by the geometrical parameters, including the shape and dimensions of the bioreactor and sparging bubble size. Hence, the smaller the bubble size is, the higher the interfacial area would be, thus enhancing oxygen transfer. The oxygen mass transfer coefficient is impacted by medium formulations, mixing, energy dissipation, exposure time, etc. For example, Shah et al.’s experimental results showed that the volumetric oxygen mass transfer coefficient (k_L_a) increased from 2 to 3.4 h^−1^ when the agitation speed was increased from 6 to 20 rpm in a 2-L gyratory motion bioreactor with an airflow rate of 0.05 vvm, which illustrates that the higher agitation speed improves the oxygen transfer efficiency [80]. The oxygen concentration gradient depends upon the difference between dissolved oxygen concentration and saturated concentration in the medium. Thus, pure oxygen in the headspace leads to the increase of this gradient and acceleration of oxygen delivery.

#### 2.3.3. Medium and Feeding Strategies

Similar to planar culture, the most common medium used for hMSC expansion in microcarrier-based bioreactors is DMEM or αMEM supplemented with FBS. Recent interest in pursuing xeno-free culture to diminish the influence of undefined animal-derived components, human serum [45] or platelet lysate [41] have been tested and demonstrated successful replacement of FBS for xeno-free microcarrier suspension cultures in bioreactors. Moreover, the potential of these cultures for therapeutic purposes has led to the development of several commercially available media specific for hMSC expansion in microcarrier-based culture systems, including MesenCultTM-XF [33], PRIME-XV MSC Expansion SFM [42], StemPro^®^ MSC SFM XenoFree [29], MSCGMTM BulletKit^TM^ [48]. Some factors are added to the growth medium for a specific purpose. For example, the addition of polymers such as 0.1% Pluronic F68 helps to protect cells through reduction of cell hydrophobicity and thus reduces shear-induced damage and facilitates hMSC attachment to microcarriers. This is especially beneficial when sparging the culture with gas and bursting bubbles [31,41,42]. Practically, the choice of medium should be optimized and dictated by the feeding strategies appropriate for individual culture systems.

As medium is the primary source of expense for hMSC production, a wise feeding plan will use the minimal volume of medium necessary to obtain a maximum cell yield without negatively impacting cell quality. The medium usage efficiency can be expressed as medium consumption per million cells (mL/10^6^ cells). Using this descriptor, the cost of medium can be compared between different studies. Batch culture is a convenient and economical reference for the beginning of process development. The advantages include ease of operation, low risk of contamination and reduced medium usage. Due to the limits of nutrition, high cell culture density cannot be achieved, thus, batch culture is not ideal for hMSC manufacturing assessments. However, batch culture is ideal for process developments which are not related to final yields, such as cell line screening, microcarrier screening, cell attachment improvement, nutrient limitation determination, bioreactor parameter optimization, batch-to-batch variation clarification and others [81,82].

Common operations for hMSC microcarrier-based culture usually implement fed-batch as the feeding strategy to avoid nutrient-related growth inhibition, including medium addition and medium change. Medium addition simultaneously replenishes nutrients and dilutes metabolic wastes. As this method lacks a draining procedure, it is not necessary to stop agitation. An added benefit of this approach is the preservation of hMSC-secreted cytokines and no cell loss during the expansion. Unfortunately, the increased working volume required by this strategy results in decreased cell production per unit volume. A partial medium change removes metabolic wastes from the bioreactor culture prior to replenishing the medium, and maintains maximum volumetric productivity. This medium removal step requires no agitation for 10 to 20 min to enable the microcarriers to settle [30,46]. The exact time required may vary due to the microcarrier density, bioreactor scale, degree of cell aggregation or other factors that impact the ability of microcarriers to settle from the medium supernatant. The frequency and portion of exchanged medium are optimized through off-line medium analyses to prevent nutrient depletion and waste accumulation. Most studies choose medium exchange volumes that are equal to or less than 50% of the working volume (Table 1). This both reduces microcarrier loss during draining and also preserves hMSC-secreted cytokines which are known to enhance expansion. In some cases, if the off-line medium analysis infers that the depletion of a specific known nutrient component will soon occur (according to the cell growth curve), a higher concentration of this component may be provided as an additive to compensate for the shortage and maintain the cell proliferation rates [32,83].

Perfusion mode (continuous medium exchange) stably provides fresh medium into the bioreactor and simultaneously pumps the spent medium out of the culture. This strategy makes it possible to obtain higher cell densities than the maximum achieved through unmodified cultures. Cunha et al. exploited the same medium usage in microcarrier-based stirred-tank bioreactors to compare the performance of the perfusion culture at an exchange volume of 20% working volume per day with a fed-batch medium change of 50% working volume per 2.5 day. Their results showed that perfusion cultures can reach higher cell concentrations at 3.7 × 10^5^ cell/mL with 14.6-fold expansion in comparison with cell concentrations of 2.9 × 10^5^ cell/mL with 11.5-fold expansion in fed-batch medium change [34]. The perfusion flow rate can be manipulated depending on the growth and off-line medium analyses to supplement nutrients as necessary with minimal medium fluctuation [29,48].

#### 2.3.4. Microcarrier Addition

In addition to medium replenishment, some studies also tested the addition of fresh, naked microcarriers as an approach to provide existing cells with more surface to expand upon and dilute cell confluency. This method depends upon efficient bead to bead cell migration. The feasibility of bead-to-bead transfer has been well demonstrated in spinners even in cultures maintained under constant agitation using various culture systems and several culture mediums [57,84]. A previous study established an approach to add fresh microcarriers into a stirred-tank bioreactor on Day 6, followed by an intermittent agitation (i.e., 15 rpm for 5 min and static for 55 min) from Day 6 to Day 9 to achieve better cell migration from beads to bead and thus maximize microcarrier colonization [34]. This could be an innovative technique to provide high cell density cultures to with increased volumetric surfaces and reduce the degree of aggregation. Even though promising, the process requires optimization to determine the ideal time frame for microcarrier addition, the amount of fresh microcarriers and overall improvement of cell bead-to-bead transfer.

### 2.4. Harvest

Once the bioreactor control system and optimized medium feeding strategies demonstrate the ability to expand hMSCs well, the next step is to determine the optimal time to harvest these cells as the final products. In contrast to planar cultures, microcarrier-based cultures present hMSC harvesting challenges. At the time of harvest, hMSCs typically occupy most of the microcarriers and the degree of microcarrier clumping is very high, likely further increasing the difficulties associated with harvesting the cells. Using amended procedures from planar culture as guides, the entire harvest process in microcarrier culture begins with cell detachment from the microcarriers. This involves steps to drain the culture medium, add and wash with PBS, drain the PBS, add dissociation buffer for incubation with agitation and quench with medium. Then, the following steps are included to efficiently remove microcarriers and to concentrate a large volume of cell suspension for the subsequent downstream bioprocesses. Each step involved in the removal of liquid from the culture system inevitably causes some loss of cells. For example, draining spent medium or wash buffer may also remove some microcarriers with adherent hMSCs and microcarrier removal and cell suspension/concentration steps both involve the loss of some cells. Detachment efficiency is likely to be another factor that reduces the total cell yield. Therefore, many approaches have been developed to improve hMSC harvest efficiencies.

The most critical part of the harvest efficiency is the cell detachment procedure. Nienow et al. reported 21 combinations of detachment in-situ in 15-mL ambrTM and 100-mL DASGIP bioreactors and 100-mL spinners with the dissociation reagents Trypsin-EDTA, TrypLE Express and Accutase to optimize the detachment efficiency in hMSC microcarrier culture [85]. In essence, their protocol takes advantage of a short-period of intense agitation with dissociation reagents to combine the chemical reaction and physical force necessary to reach the maximum detachment efficiency. The detachment efficiency can be checked at any time point by visual examination of samples under a microscope to see if the microcarriers are still attached to cells. In addition, once the cells are detached as single cells or small clusters with sizes smaller than the Kolmogorov scale of turbulence, cells will no longer suffer from the fluid shear stress [85]. After cell detachment, microcarrier removal is required to obtain a pure cell suspension. A sterile sieve can be used to filter out microcarriers. To accommodate the high volumes of large-scale preparations, single-use strainer bags are commercially available. For example, Harvestainer™ BioProcess Containers from Thermo Fisher Scientific are designed for separating microcarrier beads from the harvest broth in a closed system at different sizes, including 3 L, 12 L, 25 L and 50 L, which should be able to cover most of the scales developed thus far [86]. This involves the simple assembly of a micro-sieve bag inside a sterile containing bag to allow microcarriers to be captured in the sieve bag while detached cells will pass through it [9]. OptiCap^®^ XL 1 Capsules from EMD Millipore also provide efficient filtration to separate cells from microcarriers [34]. The sieve mesh sizes must be between microcarrier diameter and cell diameter, ranging from 60 to 100 µm (Table 1).

Regardless of the approach taken, the harvest process requires several complicated steps that must be performed in a timely manner. To reduce complexity, creative types of microcarriers have been developed to simplify the procedure and increase manufacturing speed and efficiency. Dissolvable microcarriers are an innovative approach to easily retrieve all cells easily without further filtration and have been used in liter-size bioreactor cultures [35,46,48]. Thermo-responsive microcarriers have also been tested for thermal lifting harvest [87,88]. Despite these advances, there remains room for further improvements to accelerate the harvesting process.

After microcarrier removal, the clarified hMSC suspension typically consists of a larger volume (>20 L) than that resulting from comparable planar cultures. Laboratory liter-size centrifuges lack the capacity to handle these large volumes. State-of-the-art continuous flow technologies have been developed for highly efficient cell concentration. Tangential flow filtration, also called cross flow filtration, is a design that improves permeated flow rates by setting the flow direction perpendicular to the filtration direction (as implied by the name) [34]. Many commercial products are available, including Cadence™ Single-Pass and PallSep™ Systems from Pall Corporation, Cogent M1 TFF System from Millipore, UniFlux and ÄKTA flux from Cytiva and others [89]. Another design is counterflow continuous centrifugation. These systems can retain cells at the fluidized bed thanks to the balance of the fluid flow opposite to the centrifugal force, such as kSep^®^ Systems from Sartorius Stedim or Elutra^®^ cell separation system from Terumo BCT [90]. These methods accelerate the handling time for concentrating cells from large scale volumes and reduce the time cells are exposed to the forces of centrifugation. Further optimizations to obtain maximum hMSC yields are still needed.

## 3. Sampling and Cell Counting

The efficacy of these cells as therapeutic drugs depends upon the ability to achieve high quantities of healthy viable hMSCs. Thus, methods to count cells in suspension accurately and consistently are very important. Moreover, cell counting in microcarrier-based bioreactor culture becomes more of a challenge due to the interference of microcarriers and their inherent aggregation in culture. In contrast with the consumption of nutrients and accumulation of wastes, the determination of cell number is viewed as the most direct evidence to support and confirm a successful cell expansion and thus also guides the decision and direction of future process development. In essence, cell counts are the key reference for the entire bioprocess. Before counting cells, representative samples must be collected. The sampling port location, the agitation speed at sampling, the processes to handle the sample, all may alter the counted cell number. Optimization of the sampling method is the first step and can present a challenge especially for the late stages of the cell culture, when hMSCs are growing in multi-microcarrier aggregations.

Cell counting is a relatively straight-forward process and many methods have been established for homogenous cell suspensions from planar cultures. The method is to count the cell number in a fixed volume and then to convert the number to the sample size and further to the entire culture system. When working with microcarriers in aggregation, the methods need to be modified.

**Figure 1:**
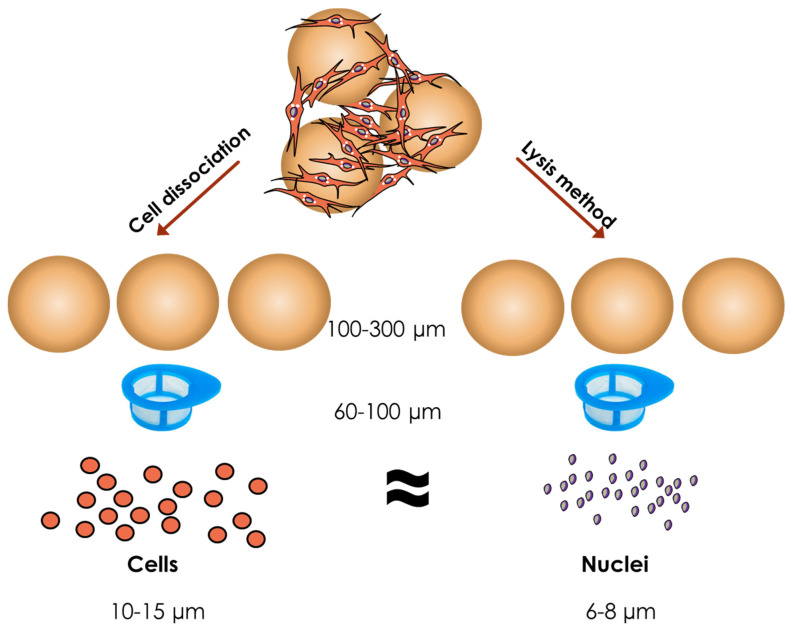
Cell dissociation and cell lysis methods. Cell counting methods for microcarrier-based bioreactor cultures.

In general, there are several ways to count cells in microcarrier culture: (1) Cell counting after cells have detached from microcarriers [26,29,37,50]; (2) Cell counting after microcarriers have dissolved [35,46,48]; (3) Nuclei counting after cells lysis with microcarriers [27,28,36]; (4) DNA quantification [18,29]; (5) Metabolic assays [34,44,47,91]. Counting methods with simple steps and high throughput are ideal and more favorable than assay-based approaches. In addition, as dissolvable microcarriers are not always feasible, cell detachment from microcarriers and cell lysis with microcarriers are often the more appropriate options (Figure 1).

Beyond manual cell counting with a hemocytometer, automated cell counters have become widely utilized in hMSC manufacturing for rapid cell number measurement in replicate samples. Even though the theory is simply to count all the cells and viability is determined by the integrity of cell membrane, different automated cell counters may yield diverse results [92]. Trypan blue enhances cell counts with viability information [27,41,50] while fluorescence dyes, such as acridine orange, calcein, DAPI, propidium iodine and others can show higher color contrast for counting live and dead cells with or without lysis buffers by automated cell and nuclear counters [17,31,36,48]. These automated counters principally analyze the images of the stained cells/nuclei and count the number of particles. Importantly, the gate should be set up according to the cell/nuclei size to eliminate irrelevant cell debris or fractured nuclei that may be present in the sample. The typical certificated range for automated counters is from 10^4^ to 10^7^ as this provides sufficient particles to count but is not so congested that the equipment may not discern individual particles. It is important to note that an automatic cell counter that works well for planar culture may be less efficient when counting cells derived from microcarrier culture due to the risk of jamming the loading tools with microcarriers [31].

## 4. Conclusions

Our previous study examined and summarized alterations in hMSC therapeutic potency attributed to the microenvironment changes that occur when switching from planar culture to microcarriers. Differentiation potentials were found to significantly increase in osteogenic and chondrogenic lineages and decrease in adipogenic lineages. Improvements in migration ability, anti-inflammatory and immunomodulatory capabilities were also noted [53]. We also acknowledged shortcomings that stem from microcarrier cultures—primarily the heterogeneity that induces from discontinuous growth surfaces and nonhomogeneous shear stress distribution within the bioreactors [53]. However, despite their limitations, microcarrier-based bioreactor systems remain the most promising and profitable approach to manufacture hMSC-based products for commercialization. This review is focused on developments in microcarrier-based bioreactors which have the most potential for use in scaled up processes. We also discuss the required bioprocessing alterations, including seed train, inoculation, expansion, harvest, and cell counting necessary for microcarrier cultures. Bioreactor parameters, such as temperature, pH, DO, mixing and feeding, are also reviewed. Finally, we detail various cell counting methods which have been applied in hMSC microcarrier cultures.

## Figures and Tables

**Table 1:** hMSC microcarrier-based bioreactor process parameters.

Cell Type	Seed Train	Vessel Type	Vessel Volume (L)	Working Volume (L)	MC Type	MC Concentration (g/L)	Cell-to-Bead Ratio	Cell Inoculation Concentration (Cells/mL)	Cell Seeding Density (Cells/cm^2^; Cells/mL)	Strategy for Cell Attachment	Action Time for Attachment (h)	Counting Method	Attachment Efficiency	Colonization Efficiency	pH	Gas Input	Mixing	Power Ratio (W/m^3^)	Feeding Method and Regime	Period (day)	Recovery Mesh Size (µm)	Ref.
hPL-MSC	Planar culture	CultiBag or Cellbag	2	0.5	CultiSpher-S	-	5	-	-	Cell were inoculated at minimum volume of medium. Gentle rocking to distribute the cells, maintaining static culture for overnight prior to addition of medium to working volume.	18	CyQUANT cell quantification assay kit for unattached cells.	90%	-	-	5% O_2_ 5% CO_2_ Air	-	-	-	7	-	[18]
hBM-MSC	Directly transfer from spinner flask MC culture	Mobius CellReady 3L	3	2	SoloHill collagen-coated	-	4.5 ^α^	30,000	5000 (seeding before transferring)	200 mL MCs with attached cells were directly transferred from spinners into the bioreactor containing 800 mL of media with fresh MCs. 25 rpm at low volume (1 L) and then increased to 40 rpm at the larger volume (2 L) on Day 3.	-	Using NC-100 after lysing the cells off the MCs.	-	-	-	-	25 rpm to 40 rpm (after Day 3)	-	50% addition	7	100	[16]
Hf-MSC	Planar culture	Biostat B-DCU	1	1	Cytodex 3	8.34	3 or 4 ^α^	100,000	4440 ^α^	50% working volume at 30 rpm (lower agitation) for 24 h. Then, add to full working volume at 50 rpm.	24	Attached cells on MCs were counted by nuclei count NC-100; Unattached cells were counted by trypan blue.	-	-	NaOH and CO_2_ gas 7.2–7.3	O_2_ CO_2_ Air	50 rpm	-	50% medium change every 2 day	8	70	[27]
hBM-MSC	Planar culture	Biostat B Plus	5	2.5	Plastic P-102L	27.8 ^α^	5	24,000	6000	Static for 18 h.	18	Attached cells on MCs were counted by NC-100 with propidium iodide.	-	-	no control (7.2 to 6.7 and 6.9)	-	75 rpm	-	50% medium change every 2 day after Day 3	9	60	[17]
hBM-MSC	Planar culture or Fresh Thawed	Mobius CellReady	3	]	-	15	-	5000	-	Low agitation at 25–35 rpm.	24	Samples were spun down at 200 rpm for 5 min and cell numbers were measured using NC-100.	60%	-	-	5% CO2	-	-	Feeding twice (Day 6 and Day 10)	12	-	[15]
hAD-MSC	Fresh thawed	UniVessel^®^ SU	2	2	ProNectin^®^ F-COATED	7.5	2.5 ^α^	7560 ^α^	2800	The 4-h attachment phase was realized in 0.7 L culture volume without stirring before the medium was filled up to 2 L.	4	Cell densities were determined using NC-100.	-	-	7.2	0.1 vvm	100–140 rpm	1.54 at 100 rpm; 2.87 at 125 rpm; 3.94 at 140 rpm	50% medium change on Day 4	7	63	[28]
		CultiBag STR	50	35	ProNectin^®^ F-COATED	7.14		7197 ^α^		After inoculation into CultiBag RM 20-L which was placed in an incubator at 37 °C, 80% humidity and 5% CO_2_, and kept stationary for 4 h. Afterwards, the MC-cell suspension was transferred into the CultiBag STR 50-L.					7.2–7.3	0.03 vvm	50 rpm	0.63		9		
hBM-MSC hAD-MSC	Directly transfer from spinner flask MC culture	Bioflo^®^ 110	1.3	0.8	SoloHill plastic	5 (in spinner)	3.1 (in spinner) ^α^	50,000 (in spinner)	3472 (in spinner) ^α^	25 rpm for 18 h followed by a non-agitation period of 6 h (in spinner).	24 (in spinner)	Samples were washed with PBS and incubated with TrypLE Express at 37 °C for 5–7 min at 650 rpm using Thermomixer confort. for detaching cells. Single cell suspension were then counted by Trypan Blue.	-	-	7.2	CO_2_ sparging; Air 5 ccm	60 rpm	-	25% medium daily	7	-	[29]
hBM-MSC	Planar culture	DASGIP (cellferm-pro)	-	0.4	SoloHill plastic	20	3.4 ^α^	27,000	3750 ^α^	40 rpm for 2 min followed by a non-agitation period of 2 h.	24	MC-cell pellet was instantly frozen at -80 ntil cell counting using CyQUANT1 Cell Proliferation Assay (Life Technologies) based onDNA.	-	-	7	-	40 rpm	-	Perfusion at 25% working volume (100 mL/day) after 3 days	11	-	
hUC-MSC	Fresh thawed	B-DCU1 2 L univesselQuad version	2	1.5	Cytode× 1	-	1.2 (in spinner) ^α^	-	1200	450 mL in static culture for orvernight. Then, medium addition to 1500 mL at 50 rpm.	24	-	50%	-	7.35	2	50 rpm	-	50% medium change twice per week	8–13 (various donors)	80	[30]
hAD-MSC	Directly transfer from shaking flask MC culture	BioBLU 5c	3.75	3.75	SoloHill collagen-coated	17	2.7 (in flask) ^α^	17,500	3000 (in flask)	3.5 L MCs with attached cells were transferred into the bioreactor at 25 rpm. After 1 h, addition of 0.25 L medium was added to reach 3.75 L working volume.	-	Cells on MCs were counted by NC-100.	-	-	7	Air, CO_2_, N_2_, O_2_ N_2_ sparging at 0.01 SLPM after Day 6	25–35 rpm after Day 6	-	50% medium change on Day 4, 8, 12 and addition of 0.5 g/L glucose on Day 15	18 (peak on Day 16)	-	[31]
hfMSC	Harvest from spinner flask MC culture	Biostat B-DCU	2	0.8 to 1.9	Cytode× 3	8 ^α^	4	100,000	4629 ^α^	-	-	Total and non-viable cell concentrations in the MC culture were determined by NC-3000.	-	-	7.2–7.3	O_2_ CO_2_ Air	60–80 rpm	-	Periodic feeding concentrated medium every 1.5 h	6	-	[32]
hBM-MSC	Planar culture	PBS-VW	-	2.2	Synthemax^®^ II	16 to 48 after Day 6	3.9 ^α^	25,000	4340 ^α^	0–6 h: 17 rpm, 1 min; off, 20 min 6 h–day 6: 17 rpm Day 6–10: 17 rpm, 5 min; off, 1 h Day 10–14: 17 rpm.	12	Total cells were briefly disrupted using 0.1 M citric acid with 1% TritonX-100 at 37 °C overnight and the nuclei were stained with 0.1% crystal violet for counting by hemocytometer.	95%	68%	7.2	-	17 rpm	0.3	50% medium change every 2.5 day after Day 5	14	-	[33]
		Biostat Qplus ST	-	0.25						0–6 h: 40 rpm, 1 min; off, 20 min 6 h–day 6: 40 rpm Day 6–10: 40 rpm, 5 min; off, 1 h Day 10–14: 45 rpm.				48%		-	40–45 rpm	Avg. 0.1–0.2 Max. 0.6–0.8				
hBM-MSC	-	Biostat Qplus stirred tank	-	0.4	Synthemax^®^II	16 to 48 after Day 6	3.9 ^α^	25,000	4340 ^α^	For the first 6 h, 200 mL at 60 rpm for 1 min and 0 rpm for 20 min. Then, another 200 mL medium was added to reach 400 mL working volume at 40–60 rpm.	6	Trypan blue; Fluorescein diacetate-propidium iodide staining; LDH assay.			7.2	0.1 vvm	40–60 rpm (from Day 6 to 9, an intermittent agitation was set at 15 rpm for 5 min and 0 rpm for 55 min)	-	50% medium change every 2.5 day after Day 5 or perfusion rate at 20% daily after Day 5	14	75	[34]
hAD-MSC	Fresh thawed	CultiBag	2	1.5	MC-2	13.63 ^α^	5.8 ^α^	31,902 ^α^	6500	MCs were incubated at 37 °C overnight before cell inoculation into 1 L shake flasks. After a 20-h static attachment, MCs with cells were transferred into the culture bag prior to addition of medium to working volume.	20	Cells were dissociated from MCs by TrypLE Select for 30 min at 37 °C and counted by NC-200.	-	-	7.3	0.05 vvm	4° and 31 rpm	Avg. 8.92 Max. 17.69	50% medium change on Day 5 after settling down MCs for 15 min	9	-	[26]
hUCM-MSC	Planar culture	Celligen 310	2.5	0.8	Cultispher^®^S	1	31.25 ^α^	25,000	-	0–24 h: 30 rpm 24–72 h: 40 rpm after 72 h: 50 rpm.	24	Samples were trypsinized to recover bead-free cell suspension (i.e., no filtration step needed).	75	-	7.3 (NaHCO_3_ and H_2_SO_4_)	N_2_ O_2_ Air	50 rpm	-	no medium change	6 (peak on Day 4)	-	[35]
hBM-MSC	Planar culture	Mobius^®^3 L	3	2.4	SoloHill collagen-coated	15	2.5 ^α^	15,000	2777 ^α^	1 L for cell attachment.	-	-	-	-	7.4–7.6	-	-	-	-	13	-	[36]
		Mobius^®^50 L	50	50						20 L at 64 rpm for 4 h.	4	Total cells were counted by nuclei using NC-100; unattached cells were counted after filtration with 100 µm sieve; attached cells were counted after unattached cell removal and PBS wash.	13% at 4h and >100% after 24 h	-	7.45 (NaHCO_3_)	1 lpmAir CO_2_ N_2_ O_2_ instead of Air after Day 4	75 rpm - 85 rpm after Day 7 - 95 rpm after Day 9 -100 rpm at final	-	Day 3 and Day 7	11	-	
hUCM-MSC	Directly transfer from spinner flask MC culture	Celligen 310	2.5	0.8	Plastic P102L	10 (in spinner)	10 (in spinner) ^α^	40,000 (in spinner)	11,000 (in spinner)	Intermittent stirring was set for 3 days: 2 min agitating at 30 rpm followed by 15 min static (in spinner).	72 (in spinner)	MCs with attached cells were treated with TrypLE and Collagenase for 7 min. Detached cells were counted by Trypan Blue exclusion method.	-	-	7.3	-	30 rpm	-	25% medium change daily after Day 5	7	100	[37]
hBM-MSC hAD-MSC	Planar culture	2 L UniVessel^®^SU	2	2	Synthemax^®^II	32 to 16 after 5 h	3.9 ^α^	25,000	4340 ^α^	50% working volume for 5 h (On:100 rpm for 1 min; Off: 0 rpm for 20 min). Addition of medium to full working volume at 100 rpm.	5	-	84%	85	7.2	0.1 vvm	100 rpm	1.54	50% medium change on Day 5	7	-	[38]
hWJ-MSC	Planar culture	B-DCU	1	0.3 to 0.48	Low density Polycaprolactone	31.3 ^α^	4	120,000	7330 ^α^	-	-	Viable cells were counted with NC-3000.	-	-	7.2	Air CO_2_ N_2_ O_2_	80 rpm	-	Medium addition to maintain glucose concentration at 0.2 g/L after Day 3	7 (peak on Day 5)	-	[39]
hUCM-MSC	Planar culture	Celligen^®^ 310	2.5	0.8	CultiSpher-S^®^	2	-	-	5000	a stirring period (50 rpm for 1 min) followed by 30 min of nostirring. After 6 h of cell adhesion, a continuous stirring was set at 50 rpm.	6	Cell number was quantified using MTT assay.	-	-	7.3 (NaHCO_3_ and H_2_SO_4_)	N_2_ O_2_ Air	50 rpm	-	50% medium change daily after Day 4	7	100	[40]
hAD-SC	Planar culture	Mobius^®^ 3-L	3	2	Corning^®^ Enhanced Attachment	15	3.6 ^α^	21,625 ^α^	4000	0.5% PL instead of 2% and supplemented with 0.1% Pluronic^®^ F68 for 24. Then, PL was added to reach 5% PL at 800 mL working volume.	24	Cells were harvested and counted using Trypan blue exclusion.	-	-	7.5	Air (27 mL/min) CO_2_	35 rpm	-	-	12	-	[41]
hBM-MSC	Planar culture	DASGIP DASbox	0.25	0.1	Plastic P-102L	13.8 ^α^	5	30,000 ^α^	6000	The culture was static for one hour and then start to agitate.	24	Total and non-viable cell concentrations in the MC culture were determined by NC-3000.	30% at sparging and 60% at overlay after 24 h	-	7.4	Air sparging 0.1VVM	115 rpm	2.56^α^	50% medium change every other day	6	-	[42]
hUCM-MSC	Planar culture	Celligen^®^310	2.5	0.8	CultiSpher-S^®^	2	-	-	5000	A stirring period (50 rpm for 1 min) followed by 30 min of no stirring. After 6 h of cell adhesion, a continuous stirring was set at 50 rpm.	6	Cell number was quantified using MTT assay.	-	-	7.3 (NaHCO_3_ and H_2_SO_4_)	N_2_ O_2_ Air	50 rpm	-	50% medium change daily after Day 4	7	-	[43]
hUCM-MSC	Directly transfer from shaking flask MC culture	Xuri™ 2/10Cellbag	2	0.6	CultiSpher-S^®^	2.1 (in spinner)	30	50,000 (in spinner)	-	0–1 h: 200 mL at 50 rpm for 1 min every 15 min 0–3 h: 200 mL at 50 rpm for 1 min every 30 min 3–9 h: 200 mL static 9–24 h: – After 24 h, the culture was transferred into cellbag and medium was added to reach 600 mL at 24 rpm 4°	24	Cells adhered on MCs was indirectly measured by MTT assay.	94.80%	-	6.8–7.4	5–10% CO_2_ Air 0.02 to 0.04 lpm after 24 h	24–48 h: 15 rpm 7° 48–168 h: 24 rpm 4° 168–216 h: 27 rpm 3° 216–240 h: 33 rpm 2°	-	-	10	-	[44]
	Planar culture					8.3 to 0.7 after 24 h		200,000	-	0–24 h: 50 mL at Static After 24 h, medium addition to reach 600 mL at 24 rpm 4°.	24		60.5%–77.8%	-			24 rpm 4°	-	-			
hAD-MSC	Planar culture	Applikon mini	500	250	SoloHill plastic	20 to 12 after Day 3	10.4 ^α^	83,333 ^α^	11,574 ^α^	0–24 h: 150 mL at 85 rpm 24–48 h: 150 mL at 95 rpm 48–72 h: 250 mL at 95 rpm.	24	Samples were washed with PBS and incubated with TrypLE Express at 37 °C for 7 min at 650 rpm using Thermomixer. Then, after quenching enzymatic activity, the cell/MC suspension was filtered using a 100 mm cell strainer for counting cells by Trypan Blue exclusion method.	22	-	7.3	N_2_ O_2_ Air	85 rpm - 95 rpm after Day 2 - 105 rpm after Day 5	-	25% medium change daily after Day 4	9 (peak on Day 7)	-	[45]
hAD-MSC	Directly transfer from spinner flask MC culture	3D FloTrix vivaSPIN	1	1	3D TableTrix	3.3 (in spinner)	2 (in spinner)	33,333 (in spinner) ^α^	1111 (in spinner) ^α^	58 cycles at 60 rpm for 5 min and 0 rpm for 20 min. Then, the culture was maintained at 60 rpm (in spinner).	24 (in spinner)	MCs attached with cells were dissolved for counting.	98 (in spinner)	-	-	5% CO_2_ 95% Air	60 rpm	-	50% medium change every other day	7	-	[46]
hUC-MSC	Planar culture	Xuri™ 2/10 Cellbag	2	0.6	CultiSpher-S^®^	8.3 to 0.7 after 24 h	16 30	204,000 to 17,000 after 24 h (high seeding) 108,000 to 9000 after 24 h (low seeding)	-	50 mL at Static for 24 h. Then, medium addition to reach 600 mL at 24 rpm 4°.	24	Cells on MCs were measured by MTT assay.	>73%	-	7–7.4	5–10% CO_2_ Air 0.02 to0.04 lpm after 24 h	24 rpm 4°	-	no medium change	7 (high seeding) 11 (low seeding)	-	[47]
hMSC	Planar culture	BioBLU 3 L	3	1	redox-sensitive beads (RS beads) regular gelatin-based beads (Reg beads)	4	-	-	-	The first 6.8 h: 0 RPM for 60 min, then 50 RPM for 10 min.	6.8	MCs with attached cells were washed with PBS and incubated with dissolution reagent in a 1:1 volume. Once dissolved, the sample was measured by NC-200.	-	-	7.2	Air CO_2_ N_2_ O_2_	55 to 100 rpm	-	Perfusion at 100% working volume per day	7	-	[48]
hUC-MSC	Directly transfer from spinner flask MC culture	Middle Scale Bioreactor BCP	0.5	0.5	Corning^®^ CellBIND^®^	-	2.7 to 4 (in spinner) ^α^	-	3000 to 4500	15 to 30 rpm after inoculation and 50 rpm on Day 2.	72	-	-	-	-	190 cc/h Air 10 cc/h CO_2_	10 to 15 rpm after transferring and 25 rpm on Day 3	-	50% medium change on Day 7 and Day 11	11	-	[49]
hUC-MSC	Planar culture	MiniBio	0.5	0.2	SphereCol^®^	5	17	40,000	22,222 ^α^	For the first 4 h, 80 rpm for 30 s, followed by 0 rpm for 30 min.	4	Cell number and viability were determined by Trypan Blue (0.4%)exclusion method after sample cell harvesting.	63%		7.2–7.4(NaHCO_3_ and H_2_SO_4_)	CO_2_ N_2_O_2_	80 to 120 rpm	-	-	5	100	[50]

Abbreviations: Microcarrier (MC), mesenchymal stem cells (MSC), human adipose tissue-derived MSC (hAD-MSC), human bone marrow-derived MSC (hBM-MSC), human placenta-derived MSC (hPL-MSC), human umbilical cord-derived MSC (hUC-MSC), human umbilical cord matrix-derived MSC (hUCM-MSC), human fetal bone marrow-derived MSC (hF-MSC), human Wharton’s jelly-derived MSC (hWJ-MSC). ^α^: The numbers were calculated based on authors’ information.
